# Investigations on Thermal Conductivity of Two-Phase WC-Co-Ni Cemented Carbides through a Novel Model and Key Experiments

**DOI:** 10.3390/ma16072915

**Published:** 2023-04-06

**Authors:** Shiyi Wen, Jing Tan, Jianzhan Long, Zhuopeng Tan, Lei Yin, Yuling Liu, Yong Du, George Kaptay

**Affiliations:** 1 School of Metallurgy and Environment, Central South University, Changsha 410083, China; 2 State Key Laboratory of Powder Metallurgy, Central South University, Changsha 410083, China; 3 State Key Laboratory of Cemented Carbide, Zhuzhou 412000, China; 4 Ganzhou Achteck Tool Technology Co., Ltd., Ganzhou 341000, China; 5 Institute of Physical Metallurgy, Metalforming and Nanotechnology, University of Miskolc, Egyetemváros, 3515 Miskolc, Hungary; 6 ELKH-ME Materials Science Research Group, Egyetemváros, 3515 Miskolc, Hungary

**Keywords:** cemented carbides, WC-Co-Ni, thermal conductivity, modeling, CALPHAD, LFA method

## Abstract

Excellent thermal conductivity is beneficial for the fast heat release during service of cemented carbides. Thus, thermal conductivity is a significant property of cemented carbides, considerably affecting their service life. Still, there is a lack of systematic investigation into the thermal conductivity of two-phase WC-Co-Ni cemented carbides. To remedy this situation, we integrated experiments and models to study its thermal conductivity varying the phase composition, temperature and WC grain size. To conduct the experiments, WC-Co-Ni samples with two-phase structure were designed via the CALPHAD (Calculation of Phase Diagrams) approach and then prepared via the liquid-phase sintering process. Key thermal conductivity measurements of these prepared samples were then taken via LFA (Laser Flash Analysis). As for modeling, the thermal conductivities of (Co, Ni) binder phase and WC hard phase were firstly evaluated through our previously developed models for single-phase solid solutions. Integrating the present key measurements and models, the values of ITR (Interface Thermal Resistance) between WC hard phase and (Co, Ni) binder phase were evaluated and thus the model to calculate thermal conductivity of two-phase WC-Co-Ni was established. Meanwhile, this model was verified to be reliable through comparing the model-evaluated thermal conductivities with the experimental data. Furthermore, using this developed model, the thermal conductivity of two-phase WC-Co-Ni varying with phase-fraction, temperature and grain size of WC was predicted, which can contribute to its design for obtaining desired thermal conductivities.

## 1. Introduction

WC-based cemented carbides are always utilized in harsh environments for military industry [1,2], aerospace [3,4], mechanical processing [5,6], metallurgy [7,8], rock drilling [9], mining tools [10] and other fields [11]. WC-Co is one of the most widely applied WC-based cemented carbides. However, the raw materials of Co are rare and are thus of high price. Therefore, partly or completely replacing Co is necessary for cost reduction [12,13,14,15], and Ni is considered to be one of the best alternative choices [12,13,14,16,17,18,19]. In such a situation, WC-Co-Ni cemented carbides have received extensive attention for replacing WC-Co cemented carbides [12,13,14,16,17,18,19].

Currently, the main investigations concerning WC-based cemented carbides are focused on mechanical properties [12,20,21,22,23,24,25,26,27]. Study concerning their thermal properties still needs to be advanced. As one of the most important thermal properties, thermal conductivity can critically affect the service life of cemented carbides. The higher the thermal conductivity, the more quickly the heat can be released during service, especially in harsh environments [18]. In such cases, the thermal fatigue behavior does not tend to occur, which can critically improve the service life. To fill the gaps in knowledge on thermal conductivity for WC-based cemented carbides, we developed models for calculating thermal conductivities for single-phase solid solutions [28] and two-phase composite materials [29] in our previous work. Coupling these models, we successfully established thermal conductivity models for calculating and predicting the thermal conductivity of WC-based systems with simple binder phases, e.g., WC-Co, WC-Ag, as well as WC-Ni systems [29,30]. Still, a model and an investigation into the thermal conductivity of the WC-based system with composite binder phases such as WC-Co-Ni have not ever been realized. It should be noted that the working temperature is one important factor for modeling the thermal conductivity. For the working temperature of WC-based cemented carbides, Casas et al. [31] found that after being exposed in air at 700 °C, the flexural strength of WC-Co drastically reduced within a short time. Liu [32] and Shi et al. [33] concluded that the intense oxidation of WC-Co occurred at temperatures higher than 700 °C. Therefore, 700 °C should be the maximum temperature of WC-Co cemented carbides for the normal service. Although no research can be found in the literature for the working temperature of the WC-Co-Ni system, it should be close to 700 °C since Ni is chemically similar to Co. Consequently, the two-phase WC-Co-Ni system is selected in this work as the target to study its thermal conductivity covering the working temperature range through the expansion of our previously developed models as well as key experiments.

Since no measured thermal conductivity of this system is available in the literature, firstly we are to use the CALPHAD (Calculation of Phase Diagrams) approach to design several WC-Co-Ni samples, which are located in the two-phase region (WC hard phase + (Co, Ni) binder phase) in the calculated phase diagram. Next, the thermal conductivity of the prepared WC-Co-Ni samples from room temperature to 1100 K will be measured, which covers the working temperature range. For modeling, thermal conductivity models for (Co, Ni) binder phase and WC hard phase are to be established through our previously developed models for single-phase solid solutions [28]. Subsequently, the key parameter, i.e., ITR of two-phase WC-Co-Ni is to be assessed. Substituting the obtained ITR, the thermal conductivity of (Co, Ni) the binder phase and the one of the WC hard phase into the thermal conductivity model for composites [29], the model for evaluating the thermal conductivity of the two-phase WC-Co-Ni is finally developed. Its reliability will be further validated through comparing the model-evaluated thermal conductivities with the experimental data. Using this developed model, the thermal conductivity for two-phase WC-Co-Ni cemented carbides along temperature, phase fraction and grain size of WC is to be predicted to extract quantitative analysis. The present work is the first attempt to model the thermal conductivity of two-phase composites with complex binder phase. It is expected that the present work can contribute to the investigations into thermal conductivity for WC-based composite materials with composite binder phases and the design with desired thermal conductivities.

## 2. Materials and Methods

**Figure 1:**
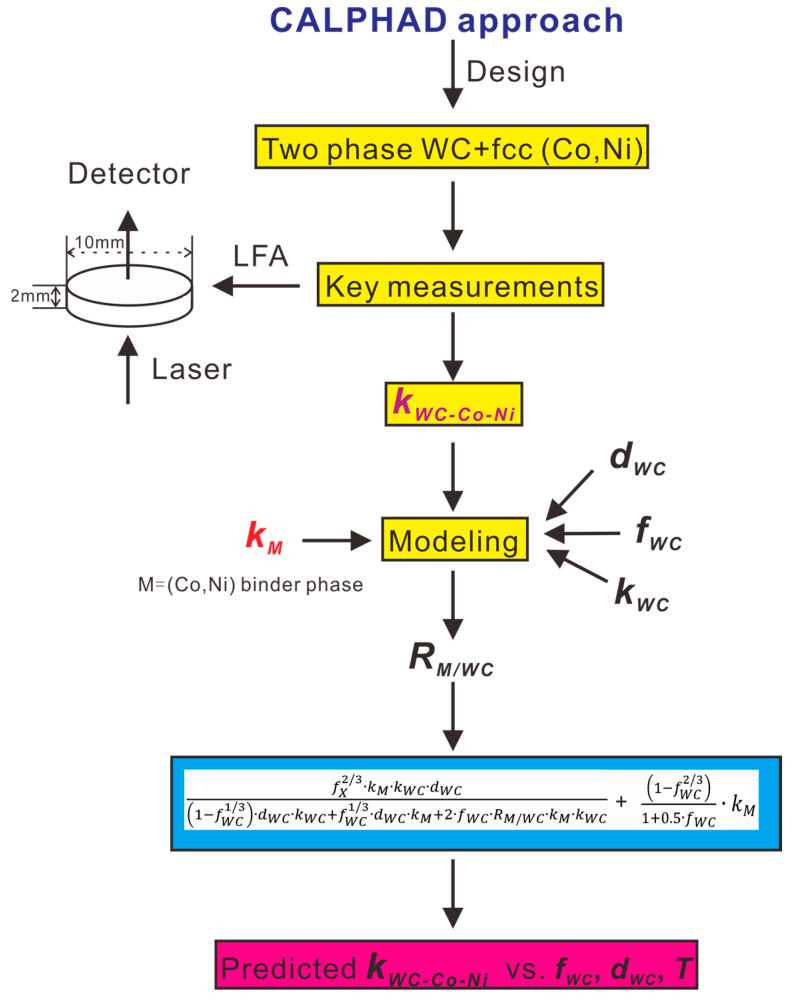
The overall strategy of the present work integrating experiments and models. *k_WC_* and *k_M_* represent thermal conductivity for WC phase and M binder phase, respectively. *d_WC_* represents grain size of WC while *f_WC_* is volume fraction of WC. *R_M/WC_* represents ITR (Interface Thermal Resistance) between the WC phase and the binder phase.

Figure 1 shows the general strategy for obtaining thermal conductivity of two-phase WC-Co-Ni varying with phase composition, temperature and WC grain size, combining key measurements and the modeling process. 

### 2.1. The Preparation of Two-Phase WC-Co-Ni Samples

**Figure 2:**
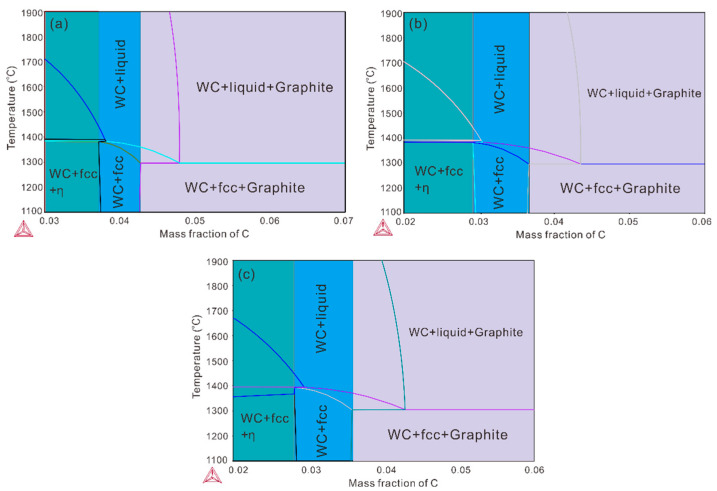
Evaluated phase diagrams by Thermo-calc software: (**a**) WC-30 wt.% (Co, Ni) (Co:Ni = 1:1); (**b**) WC-40 wt.% (Co, Ni) (Co:Ni = 1:2); and (**c**) WC-40 wt.% (Co, Ni) (Co:Ni = 1:1).

**Figure 3:**
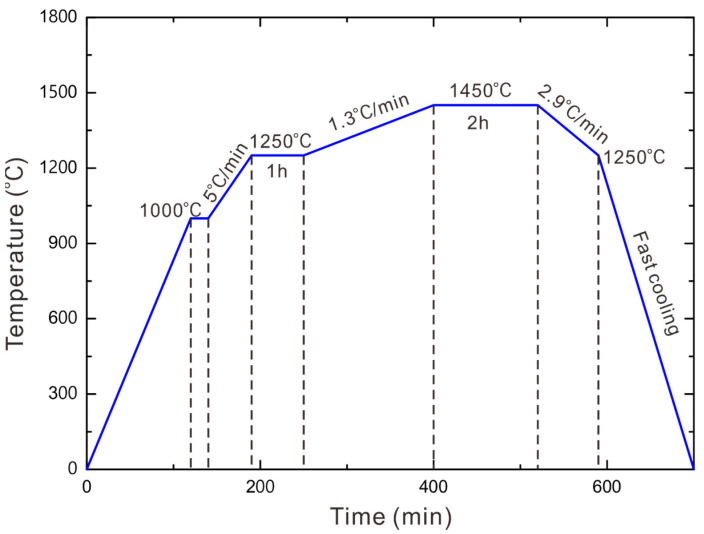
The strategy of the sintering process of WC-Co-Ni designed through thermodynamic calculations.

To prepare two-phase WC-Co-Ni samples composed of the WC phase as well as the (Co, Ni) phase, thermodynamic calculations were firstly conducted through CSUTDCC2 (Central South University Thermodynamic Database for Cemented Carbides: version 2) [34,35] input into Thermo-Calc software [36]. Figure 2a–c present the evaluated phase diagrams of WC-30 wt.% (Co, Ni) (Co: Ni = 1: 1), WC-40 wt.% (Co, Ni) (Co: Ni = 1: 2) and WC-40 wt.% (Co, Ni) (Co: Ni = 1:1) systems, respectively. According to these calculated phase diagrams, we designed the compositions for the two-phase WC-Co-Ni samples which should be located in the ‘carbon window’ (WC + fcc region). The starting WC (5.0–6.5 μm and 1.0–2.0 μm), Co (1.0–2.0 μm) and Ni (1.0–2.0 μm) powders are from Zhuzhou HaoKun Company, China, and are weighed according to the above-mentioned composition design for each sample and then mixed in a roller mill for 2 days. Cemented carbide balls are utilized as milling bodies and the weight ratio of powders to balls is 10:1, while the rotational speed is set to 320 RPM (Revolutions Per Minute). Subsequently, milled powders are dried, sieved and then put in cuboid green compacts, of which the size is Φ 15 × 15 × 5 mm. For the next liquid-phase sintering process, the strategy is designed according to the results of thermodynamic calculations, which are presented in Figure 3. Next, the compacts are sintered following the sintering strategy presented in Figure 3. As we can see from Figure 3, all the green compacts of WC-Co-Ni cemented carbides are firstly heated to 1250 °C for 1 h in vacuum (5 × 10^−5^ atm) for the outgassing process. Subsequently, in order to conduct liquid-phase sintering, they are heated to 1450 °C for 2 h. Afterwards, the cooling is continued until the furnace is shut down. All the sintered WC-Co-Ni samples are finally put into the furnace at 1200 °C for 5 days for homogenization. It should be noted that the weight loss of each WC-Co-Ni sample after sintering is less than 0.5 wt.%, and thus the chemical analysis is not performed. Finally, all the prepared samples were cut into cylinders of Φ 10.0 × 2.0 mm using wire cut electrical discharge machining and were ground.

In order to verify whether the prepared WC-Co-Ni samples contain two phases as designed, XRD (X-ray Diffraction; Bruker D8, Advanced A25, Saarbrücken, Germany) was performed. Meanwhile, SEM (Scanning Electron Microscopy; Tescan Mira 3, Brno, Czech Republic) was further utilized to observe microstructures for the obtained samples and for the subsequent assessments of WC grain size as well. Based on the SEM photos, the average area of WC grains was obtained using ImageJ software and then WC grain size could be evaluated. 

### 2.2. Measurement of Thermal Conductivity

To measure the thermal conductivity of the obtained samples, the laser flash apparatus (Netzsch LFA 457, Selb, Germany) is utilized in this work. Thermal conductivity (k, W/mK) can be calculated from:

(1) $$k=a\cdot \rho \cdot C_{p}$$

where a (m^2^/s) is the thermal diffusivity, ρ (kg/m^3^) the density and $C_{p}$(J/kgK) the heat capacity of the samples to be measured. It should be noted that thermal conductivity is not directly measured, but is calculated from the measured thermal diffusivity a, density ρ and heat capacity $C_{p}$. Thermal diffusivity can be determined by Equation (2) as [37]:

(2) $$a=0.1388\cdot \frac{l^{2}}{t_{1/2}}$$

where *l* (m) is the sample thickness, and *t*_1/2_ (s) is the time interval for the temperature of rear surface reaching 50% of the maximum one of the back surface. Density ρ of the prepared WC-Co-Ni samples is measured by using the Archimedes method. The heat capacity $C_{p}$ of the samples is described as:

(3) $$C_{p}=C_{p,ref}\cdot \frac{m_{ref}}{m}\cdot \frac{{∆T}_{ref}}{∆T}$$

where $C_{p}$ (J/kgK) is the heat capacity for the sample with mass m (kg) and measured temperature rise ∆T (K), while $C_{p,ref}$ (J/kgK) is that for the reference sample with the mass $m_{ref}$ (kg) and measured temperature variation ${∆T}_{ref}$ (K). The target sample and the reference sample are both heated by the laser flash to obtain the ∆T and ${∆T}_{ref}$, and then the heat capacity of the target sample $C_{p}$ is obtained in comparison with that of the reference sample $C_{p,ref}$, as expressed in Equation (3). 

Based on the previous work [28], the measurement uncertainty of thermal conductivity is estimated to be ±10%. 

## 3. Models

### 3.1. The Model for (Co, Ni) Binder Phase

In order to establish the model for describing thermal conductivity of two-phase WC-Co-Ni varying with the temperature, composition and WC grain size, the thermal conductivity of WC and (Co, Ni) phases should be modeled firstly. The model for the WC hard phase is directly taken from [29] while the one for (Co, Ni) is developed via the Calphad-type model [28]. The thermal conductivity *k*_M_ for the binary M (M = (Co, Ni) in the present work) binder phase can be described as [28]:

(4) $$\frac{1}{k_{M}}=\frac{x_{Co}}{k_{Co,\alpha }}+\frac{x_{Ni}}{k_{Ni,\alpha }}+x_{Co}\cdot x_{Ni}\cdot \sum_{j=0}r_{j,Co-Ni,\alpha }\cdot {\left(x_{Co}-x_{Ni}\right)}^{j}$$

where $x_{Co}$ and $x_{Ni}$ are the atomic percent of elements Co and Ni. $k_{Co,\alpha }$ (W/mK) and $k_{Ni,\alpha }$ (W/mK) are the thermal conductivities of pure Co and Ni in the α state, respectively. $r_{j,Co-Ni,\alpha }$ (mK/W) is the *j*th order thermal resistivity interaction parameter for phase α between components Co and Ni. 

### 3.2. The Model for Two-Phase Composites

Equations (5)–(7) show our recently developed thermal conductivity model for two-phase composite materials [29], which has been validated for two-phase WC-Co [29], WC-Ag [29] and WC-Ni [30] systems.

(5) $$k_{HM}=k_{path1}+k_{path2}$$

(6) $$k_{path1}=\frac{\left(1-f_{WC}^{\frac{2}{3}}\right)}{1+0.5\cdot f_{WC}}\cdot k_{M}$$

(7) $$k_{path2}=\frac{f_{X}^{2/3}\cdot k_{M}\cdot k_{WC}{\cdot d}_{WC}}{\left(1-f_{WC}^{1/3}\right)\cdot d_{WC}\cdot k_{WC}+f_{WC}^{1/3}\cdot d_{WC}\cdot k_{M}+2\cdot f_{WC}\cdot R_{M/WC}{\cdot k}_{M}\cdot k_{WC}}$$

where $k_{HM}$ (W/mK) represents the thermal conductivity of two-phase composite materials (i.e., WC + (Co, Ni) phases in the present work), $k_{WC}$ (W/mK) the thermal conductivity of the WC hard phase, $f_{WC}$ the volume fraction of the WC grains in the composites, $R_{M/WC}$ (m^2^K/W) the ITR between M and WC phases and $d_{WC}$(m) the WC grain size. $k_{path1}$ is the thermal conductivity resulting from the heat conduction through the straight path across the phase interfaces, while $k_{path2}$ is the one from the heat conduction through the continuous binder phase. Next, combining parallel and series rules, $k_{path1}$ and $k_{path2}$ can be expressed as Equation (6) and Equation (7), respectively. For the model’s development in detail, one can refer to [29]. It should be noted that $k_{M}$ and $k_{WC}$ can be obtained as described in Section 3.1. The limitation of this model is that the key parameter $R_{M/WC}$ in Equation (7) is difficult to obtain, and is set to be the only parameter to be fitted by substituting the key thermal conductivity measurements into Equation (7). Therefore, it is necessary to conduct a certain amount of thermal conductivity measurements to fit the $R_{M/WC}$.

## 4. Results and Discussion

### 4.1. Experimental Results

**Figure 4:**
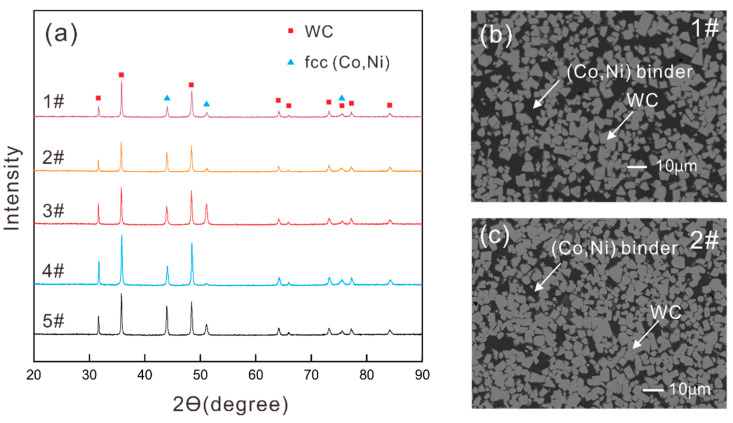
(**a**) XRD patterns of prepared WC-Co-Ni samples; SEM observations for (**b**) sample 1#; (**c**) sample 2#.

Phase compositions of these five prepared WC-Co-Ni samples are shown in Table 1. Figure 4a shows the XRD results for all the samples and Figure 4b,c present the representative microstructure observations via SEM for samples 1# and 2#. From Figure 4, the two-phase structure of the prepared WC-Co-Ni samples can be verified, laying solid foundations for the present work. The WC grain sizes obtained via the SEM figures are also included in Table 1. It should be noted that *f*_WC_ was obtained via the molar volume and weight percent of each phase.

**Figure 5:**
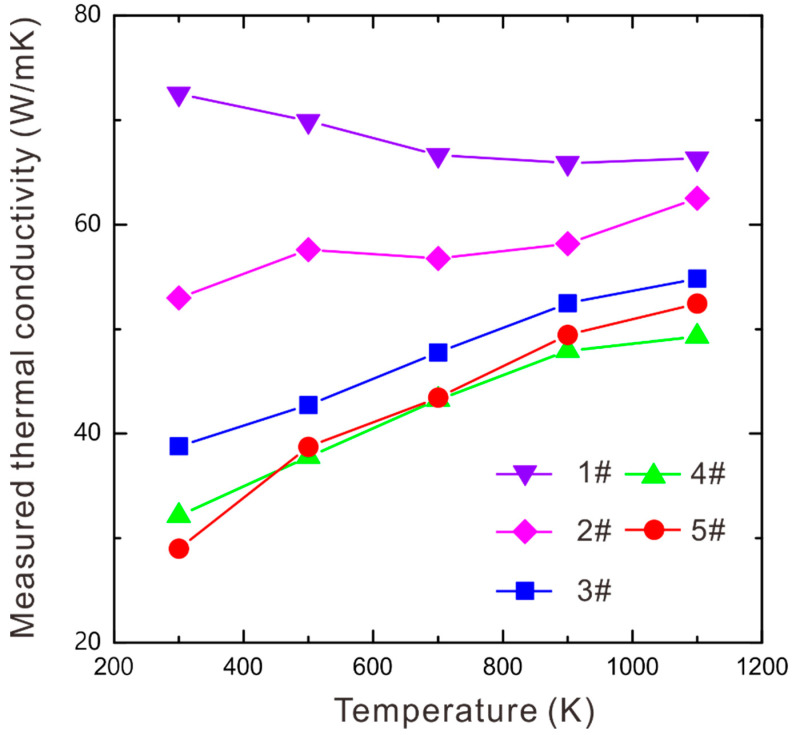
Temperature dependence of the measured thermal conductivity of the two-phase WC-Co-Ni samples prepared here.

The results of the LFA-measured thermal conductivities of every prepared WC-Co-Ni sample at 300, 500, 700, 900 and 1100 K are also listed in Table 1. To present the variation tendency of the measured thermal conductivity along with temperature, Figure 5 is plotted. From Figure 5, one can see that the thermal conductivity of sample 1# decreases with the increase in temperature, while the thermal conductivities of samples 2#, 3#, 4# and 5# increase with the increasing temperature. This is consistent with previous investigations [29,38] finding that the temperature dependence of the thermal conductivity of two-phase composite materials varies with the composition and WC grain size. For samples 3#, 4# and 5#, the ITR is the dominating factor influencing thermal conductivity. The reason is because their grain sizes are small, leading to the extremely large interfacial area between the two phases. In this case, the decreasing tendency of ITR vs. temperature results in the increasing tendency of thermal conductivity vs. temperature. While for samples with coarse WC grains, the thermal conductivity of each phase becomes the main factor. Especially when the WC volume fraction is large, the drastic decreasing tendency of thermal conductivity of WC (see Figure 4a in [29]) leads to the thermal conductivity of WC-Co-Ni decreasing with temperature. In this case, it is reasonable that thermal conductivity of sample 1# decreases with temperature. The situation of sample 2# is complex because the volume fraction of WC is not large, and the WC grains are coarse. However, from the measurements, it can be concluded that the ITR influences total thermal conductivity a little bit more than the thermal conductivity of the WC phase. Moreover, it is also found from Figure 5 that the measured thermal conductivities of sample 4# are close to those of sample 5#. It should be noted that the only difference between sample 4# and sample 5# is the composition of the (Co, Ni) binder phase, as presented in Table 1. Therefore, it can be inferred that the composition of the (Co, Ni) binder phase has little influence on the total thermal conductivity of two-phase WC-Co-Ni, which makes sense since element Co is chemically similar to element Ni.

### 4.2. Establishment of Thermal Conductivity Model for Two-Phase WC-Co-Ni System

**Figure 6:**
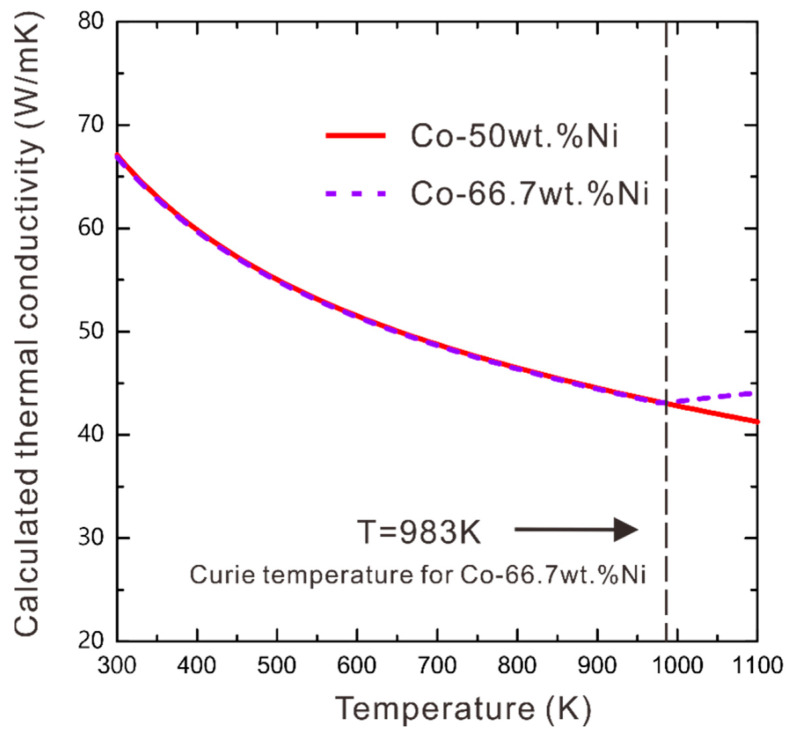
Model-evaluated thermal conductivity of Co-50wt.%Ni and Co-66.7wt.%Ni solid solutions along with the temperature.

As described in Section 3, in order to model thermal conductivity for two-phase WC-Co-Ni, the models for the WC hard phase and the (Co, Ni) binder phase should be established first. Previously, we developed the thermal conductivity model of the binder phase (Co, Ni) solid solution [28] and the hard phase WC [29]. The developed model parameters to evaluate the thermal conductivity for the WC and (Co, Ni) phases are presented in Table 2. In this work, compositions of the binder phase for samples 1#, 2#, 3# and 4# are all Co-50wt.%Ni, while the one for sample 5# is Co-67wt.%Ni. In the next modeling process of thermal conductivity for two-phase WC-Co-Ni cemented carbides, we use measured thermal conductivities of samples 1#–4# since they have the same composition of (Co, Ni) binder phase. Moreover, the measured thermal conductivity of sample 5# will be utilized for testing the prediction ability of this developed model. As important parameters for modeling thermal conductivity of two-phase WC-Co-Ni cemented carbides, the thermal conductivity of Co-50wt.%Ni and Co-67wt.%Ni binder phases were calculated via the previous model [28], as presented in Figure 6. It is observed from Figure 6 that below the Curie temperature of Co-67wt.%Ni, i.e., 973K, there is little deviation between thermal conductivity for Co-50wt.%Ni and Co-67wt.%Ni since both of them are stable in the fcc-ferromagnetic state. However, above 973 K, Co-50wt.%Ni is still an fcc-ferromagnetic phase, while Co-67wt.%Ni changes into an fcc-paramagnetic phase, leading to the difference in thermal conductivities between these two solid solutions. Nevertheless, at 1100 K, the thermal conductivity of Co-50wt.%Ni, i.e., 41.3 (W/mK) is close to that of Co-67wt.%Ni, i.e., 44.1 (W/mK). This modeling result verified the experimental observation as mentioned in Section 4.1 that the thermal conductivities measured for sample 4# are close to those for sample 5#. 

**Figure 7:**
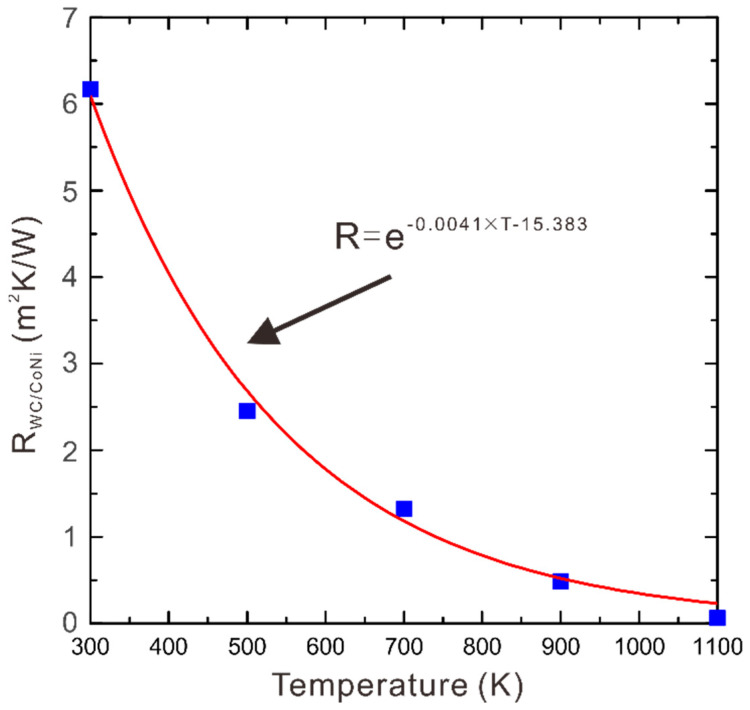
The calculated ITR in WC/(Co, Ni) interface along with the temperature.

In order to use the model presented in Section 3.2 to evaluate the thermal conductivity of the two-phase WC-Co-Ni system, the only parameter that needs to be fitted in Equation (7) is the ITR. Other parameters, i.e., temperature, WC grain size and composition are listed in Table 1, while *k*_WC_ and *k*_Co-Ni_ are shown in Table 2. Therefore, substituting the measured thermal conductivities shown in Table 1 and the known parameters into Equations (5)–(7), ITR between the WC and (Co, Ni) phases is assessed. The ITR result is presented in Figure 7, where symbols are the results at each temperature while the fitted line shows its temperature dependence. Finally, substituting all the model parameters listed in Table 2 into Equations (5)–(7), the model for evaluating thermal conductivity of two-phase WC-Co-Ni against temperature, composition and grain size of WC is developed.

**Figure 8:**
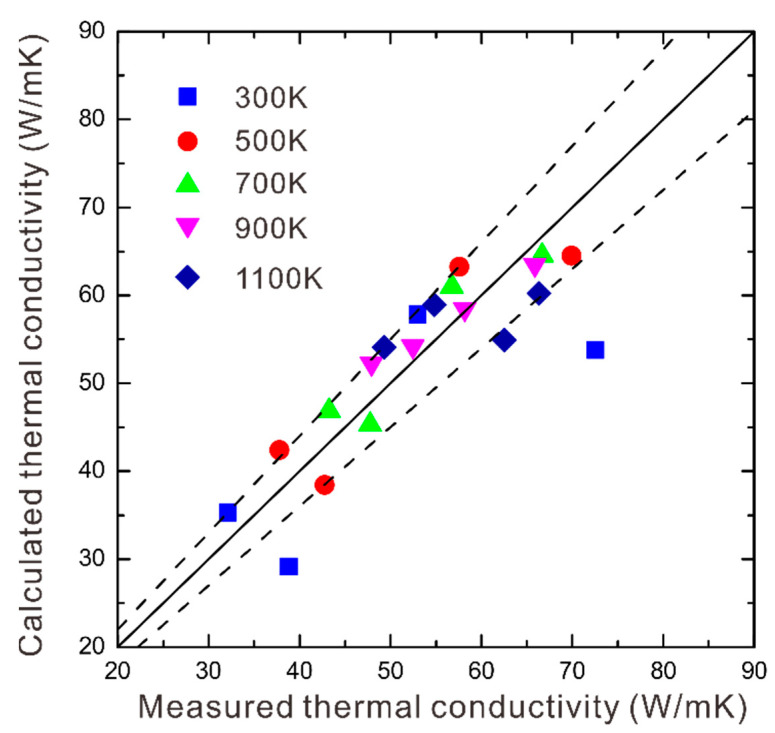
The comparison between the thermal conductivities evaluated via this developed model and the LFA-measured ones.

Before using this developed model to predict thermal conductivity for two-phase WC-Co-Ni, its reliability should be validated. Thus, we made comparisons between the measured thermal conductivities for samples 1#–4# and the calculated ones by the presently established model. The comparisons are shown in Figure 8, where the dashed lines demonstrate the acceptable experimental error [28], i.e., ±10%. From Figure 8, we can see that most of them are within or close to the error lines, while 2 out of the 20 data points exceed the dashed lines. Furthermore, supposing that the parameter *R_(Co,Ni)/WC_* is independent on the composition of the binder (Co, Ni) phase, we utilized the presently developed model to predict the thermal conductivity of sample 5#, which also shows good agreement with those measured as listed in Table 1. Therefore, the presently established model is considered to be reasonable according to the above comparisons between the measured thermal conductivities and calculated ones.

**Figure 9:**
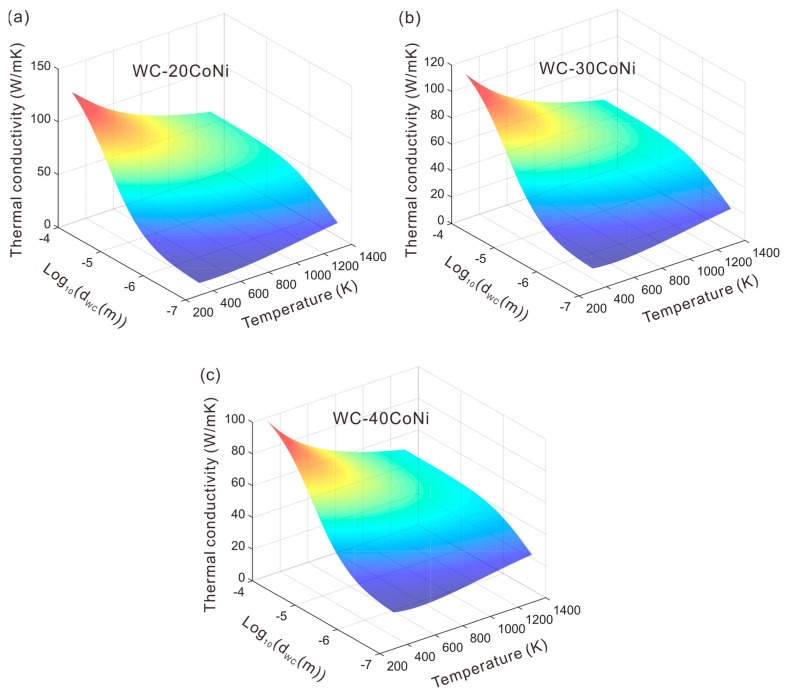
The model-predicted thermal conductivity vs. temperature and WC grain size at different phase compositions of: (**a**) WC-20 wt.% (Co, Ni); (**b**) WC-30 wt.% (Co, Ni) and (**c**) WC-40 wt.% (Co, Ni). The composition of (Co,Ni) binder phase is Co–50wt.%Ni.

Subsequently, the established model was applied to the predictions of thermal conductivity of two-phase WC-Co-Ni cemented carbides vs. temperature, WC grain size and composition. Figure 9 presents the predicted thermal conductivity vs. temperature and WC grain size at different phase compositions. From Figure 9, it can be seen that at each phase composition, thermal conductivity of two-phase WC-Co-Ni increases with the increase in WC grain size. This phenomenon is reasonable since the interfacial area between WC phase and (Co, Ni) phase decreases with the increase in WC grain size, which can accelerate the thermal conduction considerably. While for the temperature dependence of the thermal conductivity, it is found from Figure 9 that when WC grains are fine, the thermal conductivity increases with the increasing temperature. However, when WC grains are coarse, the thermal conductivity decreases with the increasing temperature. This fact was also found and analyzed in our previous work for the similar two-phase cemented carbides, i.e., WC-Co [29], WC-Ag [29] and WC-Ni [30]. When WC grains are fine, the extremely large total interfacial area between the two phases lead to ITR becoming the main factor influencing the thermal conductivity. Thus, the decreasing tendency of ITR vs. temperature is the main reason for the increasing tendency of thermal conductivity vs. temperature. When WC grains are coarse, the dominating factors are the thermal conductivities of the two phases. In this case, the decreasing tendency of thermal conductivity of each phase vs. temperature results in the decreasing tendency of total thermal conductivity vs. temperature. It is worth mentioning that with the presently established model, one could easily predict thermal conductivity for two-phase WC-Co-Ni cemented carbides at any temperature, phase composition and WC grain size according to the requirements of practical applications, which will critically contribute to designing cemented carbides with the desired thermal conductivity and also simulating the precise temperature-field during service.

## 5. Conclusions

- In total, five WC-Co-Ni samples were prepared based on the thermodynamic calculations and liquid sintering experiments. All the samples were verified to be two-phase WC + fcc (Co, Ni) through XRD and SEM measurements. Additionally, thermal conductivity values of these samples were obtained from the measured thermal diffusivity, heat capacity and density via the LFA method.
- The model for evaluating and predicting thermal conductivity for two-phase WC-Co-Ni was established, and its reliability was validated through key thermal conductivity measurements.
- Using this developed model, the thermal conductivity varying with temperature, phase fraction and WC grain size of two-phase WC-Co-Ni was predicted. It is concluded that thermal conductivity of two-phase WC-Co-Ni increases with the increase in WC composition.
- When WC grains are fine, thermal conductivity of WC-Co-Ni is observed to increase with temperature. However, when WC grains are coarse, thermal conductivity decreases with the increase in temperature.

One limitation of the present modeling process is that the key parameter ITR needs to be fitted from measured thermal conductivities. Despite this limitation, the present work provides one effective strategy for the first time to calculate thermal conductivity of two-phase cemented carbides with complex binder phase. 

## Figures and Tables

**Table 1:** Measured thermal conductivity for each prepared two-phase WC-Co-Ni sample, in comparison with the model-evaluated ones.

Sample	*f*_WC_	Temperature (K)	WC Grain Size (μm)	Thermal Conductivity (W/mK)	
				LFA-Measured	Model-Evaluated
1# WC-30wt.%(Co, Ni) ^a^	0.56973	300	6.0	72.515	53.794
	0.56973	500	6.0	69.899	64.530
	0.56973	700	6.0	66.676	64.524
	0.56973	900	6.0	65.871	63.484
	0.56973	1100	6.0	66.324	60.242
2# WC-40wt.%(Co, Ni) ^a^	0.45982	300	6.0	52.979	57.824
	0.45982	500	6.0	57.581	63.253
	0.45982	700	6.0	56.748	60.981
	0.45982	900	6.0	58.184	58.473
	0.45982	1100	6.0	62.530	54.936
3# WC-30wt.%(Co, Ni) ^a^	0.56973	300	1.5	38.790	29.178
	0.56973	500	1.5	42.752	38.457
	0.56973	700	1.5	47.752	45.281
	0.56973	900	1.5	52.488	54.226
	0.56973	1100	1.5	54.841	58.938
4# WC-40wt.%(Co, Ni) ^a^	0.45982	300	1.5	32.133	35.284
	0.45982	500	1.5	37.791	42.427
	0.45982	700	1.5	43.230	46.866
	0.45982	900	1.5	47.916	52.249
	0.45982	1100	1.5	49.291	54.106
5# WC-40wt.%(Co, Ni) ^b^	0.46015	300	1.5	28.980	35.196
	0.46015	500	1.5	38.735	42.374
	0.46015	700	1.5	43.441	46.765
	0.46015	900	1.5	49.454	52.195
	0.46015	1100	1.5	52.457	56.031

^a^ Composition of (Co, Ni) binder phase is Co–50wt.%Ni; ^b^ Composition of (Co, Ni) binder phase is Co–66.7wt.%Ni.

**Table 2:** The evaluated parameters in Equations (6) and (7).

Parameter	T, K	Equation	Reference
*k*_WC_, W/mK	300–1100	$8052\cdot T^{-0.645}$	[29]
*k*_Ni_, W/mK	300–627	$67.91-0.0367\cdot T+\frac{9727}{T}$	[28]
*k*_Ni_, W/mK	627–1100	$65.12+0.0122\cdot T-\frac{9180}{T}$	[28]
*k*_Co_, W/mK	300–695	$97.75-0.0836\cdot T+\frac{7284}{T}$	[28]
*k*_Co_, W/mK	695–1100	$46.21-0.00918\cdot T-\frac{12705}{T}$	[28]
$r_{0,Co-Ni,fcc-fer},$ (mK/W)	300–1100	0.013	[28]
$r_{1,Co-Ni,fcc-fer},$ (mK/W)	300–1100	−0.0055	[28]
$r_{0,Co-Ni,fcc-par},$ (mK/W)	300–1100	0.038	[28]
R(Co,Ni)/WC, m^2^K/W	300–1100	$e^{-0.0041\times T-15.383}$	This work

## Data Availability

Data will be available on request.
